# Stress-induced expression is enriched for evolutionarily young genes in diverse budding yeasts

**DOI:** 10.1038/s41467-020-16073-3

**Published:** 2020-05-01

**Authors:** Tyler W. Doughty, Iván Domenzain, Aaron Millan-Oropeza, Noemi Montini, Philip A. de Groot, Rui Pereira, Jens Nielsen, Céline Henry, Jean-Marc G. Daran, Verena Siewers, John P. Morrissey

**Affiliations:** 1 Department of Biology and Biological Engineering, Chalmers University of Technology, SE-41296 Gothenburg, Sweden; 2 Novo Nordisk Foundation Center for Biosustainability, Chalmers University of Technology, SE-41296 Gothenburg, Sweden; 3 Plateforme d’Analyse Protéomique Paris Sud-Ouest (PAPPSO), INRAE, MICALIS Institute, Université Paris-Saclay, 78350 Jouy-en-Josas, France; 4 School of Microbiology, Environmental Research Institute and APC Microbiome Ireland, University College Cork, Cork, T12YN60 Ireland; 5 Department of Biotechnology, Delft University of Technology, Van der Maasweg 9, 2629 HZ Delft, The Netherlands

**Keywords:** Industrial microbiology, Applied microbiology, Systems analysis

## Abstract

The Saccharomycotina subphylum (budding yeasts) spans 400 million years of evolution and includes species that thrive in diverse environments. To study niche-adaptation, we identify changes in gene expression in three divergent yeasts grown in the presence of various stressors. Duplicated and non-conserved genes are significantly more likely to respond to stress than genes that are conserved as single-copy orthologs. Next, we develop a sorting method that considers evolutionary origin and duplication timing to assign an evolutionary age to each gene. Subsequent analysis reveals that genes that emerged in recent evolutionary time are enriched amongst stress-responsive genes for each species. This gene expression pattern suggests that budding yeasts share a stress adaptation mechanism, whereby selective pressure leads to functionalization of young genes to improve growth in adverse conditions. Further characterization of young genes from species that thrive in harsh environments can inform the design of more robust strains for biotechnology.

## Abstract (web summary)

Fermentation parameters of industrial processes are often not the ideal growth conditions for industrial microbes. Here, the authors reveal that young genes are more responsive to environmental stress than ancient genes using a new gene age assignment method and provide targeted genes for metabolic engineering.

## Introduction

Yeasts in the Saccharomycotina subphylum, (budding yeasts), have proven to be useful platforms for the production of ethanol, flavors, nutritional supplements, biopharmaceuticals, as well as other valuable chemicals^1–3^. At present, industrial production using budding yeasts is dominated by the extensively characterized species *Saccharomyces cerevisiae*. *S. cerevisiae* exhibits common budding yeast phenotypes (e.g., efficient growth on some simple sugars) as well as a less common adaptation amongst budding yeasts, high ethanol tolerance^4^. Together, these traits enable cost-effective production of 100 billion liters of ethanol annually using *S. cerevisiae* as a platform^1^. Other budding yeasts have adaptations that make them well-suited for production of specific biomolecules, something that is possible due to the improved strain engineering capacity following the emergence of CRISPR/Cas9^5,6^. Examples are *Yarrowia lipolytica*, which evolved to tolerate hydrophobic environments and can produce high-yields of fatty acids^7,8^, and *Kluyveromyces marxianus*, whose thermotolerance is a beneficial feature for industrial processes^6,9^. Despite progress in sequencing genomes and phenotypic characterization of these and many other yeast species, the genes that underpin adaptation to cope with harsh conditions remain enigmatic.

For the species above, adaptations to natural environments enable robustness in industrial biotechnology processes. Understanding the genes that influence these and other exceptional stress tolerances would enable the engineering of more robust industrial strains, thereby reducing process costs and increasing yields^10,11^. Although studies that sought to characterize stress tolerances in *S. cerevisiae* have elucidated mechanisms that influence robustness^10,12,13^, engineering more robust *S. cerevisiae* strains without physiological trade-offs remains challenging^9^. One complication is that stress exposure often results in hundreds of significant transcriptional changes^13,14^, most of which do not correlate with single gene deletion changes in robustness^11^. These results suggest that multiple genes from different gene families may contribute additively to robustness and/or that stress genes may exist as duplicates, as is the case for antifreeze protein genes in artic yeasts^15^. Thus, researchers have employed systems biology to characterize the transcriptome and/or proteome-wide stress-induced changes^13,14,16–18^. These approaches have identified biological processes that exhibit altered expression in response to stress exposure, which builds upon and relates to previous research into gene functions (e.g., GO term enrichment analysis). These associations are possible due to extensive annotations of *S. cerevisiae* genes that result from decades of experimental analyses^19^. For most other yeast species, the majority of gene functional information is acquired second hand via homology search tools. This paradigm results in a large portion of genes of unknown function, which is especially large for species that are phylogenetically distant from extensively characterized species like *S. cerevisiae*^20^. These uncharacterized genes are difficult to integrate into omics analyses like GO term enrichment, as they do not have a known function or localization. Because of this, gene functional analysis of poorly characterized species is restricted to conserved genes, which may not be the only genes that influence stress-tolerance phenotypes. Currently, hundreds of whole genome sequences are available from diverse budding yeasts^21^, including several species that are known to exhibit extreme stress tolerances^22^, but many of the causative genes that enable yeast stress tolerances remain elusive.

Here, we analyze stress conditions to assess gene expression changes after stress adaptation in three diverse budding yeast species, one of which is well characterized (*S. cerevisiae*), and two that are less-well-characterized (*K. marxianus* and *Y. lipolytica*). The goal of this analysis is to identify common systems-level trends that are shared between each species stress responses. This analysis discovers that each organism displays a consistent response at the level of gene expression that is characterized by the enrichment of stress responsive genes amongst certain categories: namely, genes of unknown function and recently (in evolutionary time) duplicated and taxonomically restricted genes (young genes). The findings of this work suggest an evolutionary mechanism that is biased for stress tolerance functionalization and stress-induced expression of young genes. We propose that the gene sorting method we developed provides a path forward for more rapid identification of stress response genes in environmentally robust yeast, thereby accelerating understanding of niche adaption in budding yeasts.

## Results

### Conserved category enrichment of stress responsive genes

**Fig. 1:**
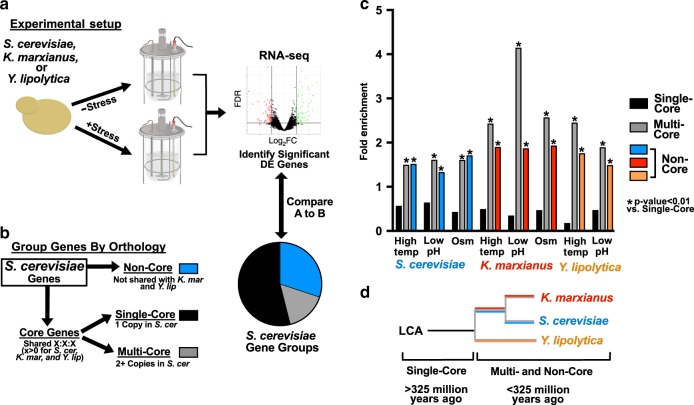
Stress adaptation responsive genes are enriched for duplicated and non-conserved genes. **a** *S. cerevisiae*, *K. marxianus*, and *Y. lipolytica* were cultivated in chemostats in standard conditions or in the presence of stress (elevated temperature, low pH, or KCl). RNAseq was performed followed by differential expression analysis. **b** The protein-coding genes of each organism were compared to infer orthology using OrthoFinder. The resulting gene groups for *S. cerevisiae* are shown, with single-copy orthologous genes (Single-Core [black]), multi-copy orthologous genes (Multi-Core [gray]), and genes that were not shared (Non-Core [blue]). **c** The number of differentially expressed (log_2_FC > 1, FDR < 0.01) mRNAs were divided by the total number of detected mRNAs inside of each ortholog group. Values were normalized to the overall DE gene # divided by the total genes measured, *p*-values were calculated using a two-sided Fisher’s exact test. **d** A simplified phylogenetic tree. Single Core orthologs are predicted to originate from a Last Common Ancestor >325 million years ago. Multi- and Non-Core Genes are predicted to have duplicated or arisen de novo <325 million years ago.

In this work, *S. cerevisiae, K. marxianus*, and *Y. lipolytica* were exposed to stress conditions that are present in natural environments, such as those caused by environmental temperature variation and growth on sugar-rich or acidic substrates^22^. These stress responses are also industrially-relevant, as they are caused by feedstocks (high osmotic pressure and low pH) or process conditions (elevated temperatures) during industrial fermentations^11^. Characterizing stress responses in these species is valuable due to their phylogenetic diversity, which spans much of the Saccharomycotina subphylum^21^. To minimize noise caused by variable growth rate^23^, experiments were carried out in steady-state chemostats at a fixed growth rate under standard and stress conditions. This experimental setup allows strains to adjust to the conditions imposed by sub-lethal stress before sampling and analysis. Transcriptomic changes that occurred in response these stress conditions were identified via differential expression analysis (Fig. 1a).

To understand the function of stress responsive genes, biological process annotations were acquired from Ensembl (*S. cerevisiae*) or identified using BLAST2GO^20^ for (*K. marxianus* and *Y. lipolytica*). BLAST2GO annotated gene functions to otherwise unknown genes based on homology to an experimentally characterized gene. This process failed to annotate 20% and 38% of the mRNAs measured by RNAseq in this study for *K. marxianus* and *Y. lipolytica*, respectively (Supplementary Fig. 1A). The lower frequency of gene annotation for *Y. lipolytica* was expected, since this species is not closely related to extensively characterized yeasts^21^. Comparison of gene annotations and differential gene expression showed a higher percentage of genes of unknown function that were stress responsive than would be expected. For example, 38% of all protein-coding genes measured in this study for *Y. lipolytica* lacked a functional annotation, while 50% of stress responsive genes were genes of unknown function (Supplementary Fig. 1B).

This high proportion of stress-responsive genes of unknown function suggested that the most broadly conserved genes, which often have functional annotations, might be under-represented amongst the stress responses. To assess this, orthologous proteins shared between the three yeast species were inferred using OrthoFinder, which enables proteome-wide matching based on amino-acid sequence and chain length similarity in order to predict proteins that descend from a common ancestor^24^. To assess the fidelity of ortholog predictions, protein complexes and enzymatic processes that were previously characterized as conserved amongst budding yeasts as single-copy genes were searched for amongst orthology inference results^25^. This analysis found that orthology inference identified the majority of the expected complex members and enzymes as orthologs (Supplementary Fig. 2B), which supports the high fidelity of OrthoFinder predictions that was observed previously^24^. The results of the orthology inference analysis were used to divide each protein into one of three classes, single-core orthologous, multi-core orthologous, and non-orthologous. These proteins were matched to their corresponding genes for comparison to RNAseq differential expression. Gene sorting examples are shown in Supplementary Fig. 2A and the complete lists of genes for *S. cerevisiae, K. marxianus* and *Y. lipolytica* are in Supplementary Data 1, Supplementary Data 2 and Supplementary Data 3, respectively.

The results of orthology inference for *S. cerevisiae* are shown in Fig. 1b as an example. Each measured protein-coding gene from *S. cerevisiae* was identified as either (1) present as a single-copy gene with an ortholog in *K. marxianus* and *Y. lipolytica* (black Single-Core), (2) present as a duplicated gene with an ortholog in *K. marxianus* and *Y. lipolytica* (gray Multi-Core), or (3) lacking an ortholog in *K. marxianus* or *Y. lipolytica* (color Non-Core). The resulting groups were compared to the observed differentially expressed (DE) genes, which showed that multi-core and non-core genes were significantly enriched amongst DE genes in each stress condition tested (Fig. 1c). The same gene sorting regime shows that *K. marxianus* and *Y. lipolytica* exhibited similar DE gene enrichment for the multi-core and non-core gene groups (Fig. 1c and Supplementary Fig. 3A). Similar results were found amongst proteomics measurements for some stress conditions (Supplementary Methods 2–5), but this analysis was hindered by low detection of non-core proteins (Supplementary Fig. 3C).

The phenomenon depicted in Fig. 1C shows that single-core genes, which are predicted to have descended from a last common ancestor between the three yeast species (approximately 325 million years ago^21^), were under-represented amongst stress responsive genes for each stress and each organism. In contrast, genes that have duplicated or emerged in more recent evolutionary time were enriched amongst stress responsive genes. These observations suggest that evolutionary events may predict differential expression amongst these diverse yeast species (Fig. 1d).

### *S. cerevisiae* stress response is enriched for young genes

The results in Fig. 1 suggested a relationship between the genes that exhibit differential expression in response to stress and evolutionary events, like de novo gene emergence and gene duplication. Further characterization of this relationship could aid in understanding stress gene evolution and could help to predict genes that enable stress tolerance. Thus, we sought to test this relationship more stringently by dividing the protein-coding genes of *S. cerevisiae* into more precise groups that collectively represent a broad swath of eukaryotic evolution. The resulting groups are referred to as gene age groups, which were determined by ortholog presence at shared copy number in common ancestors that date from over 400 million years ago to 20 million years ago^21^. A similar approach, phylostratigraphy, divides genes into groups based on homology and has been used to infer gene origination events to identify periods in evolution that correlate with adaptive events^26^. However, the results in Fig. 1c indicated that an analysis procedure that considers both gene origin timing (like phylostratigraphy) and gene duplication timing could provide insights into stress responsive gene expression.

Gene grouping based on gene age was assessed using OrthoFinder^24^ and is described in detail in Supplementary Method 1. Briefly, all *S. cerevisiae* genes were divided into three initial subsets; (1) fixed duplicates from the whole-genome duplication (WGD)^27^, (2) genes that are present as single-copy genes, and (3) duplicate genes that arose outside of the whole-genome duplication (non-WGD) (Supplementary Fig. 4A). Ortholog inference was used to sort each of the 4351 single-copy genes into a single bin based on the most distant ancestor with an orthologous gene using the hierarchal approach shown in Supplementary Fig. 4C. The multi-copy non-WGD gene groups were sorted by the presence of orthologous genes with the same copy number in a bottom-up approach in order to trace the relative timing of gene duplication events (Supplementary Fig. 4D). Finally, genes that were duplicated during the whole-genome duplication were grouped together. This sorting method matched each protein coding gene from *S. cerevisiae* to a single group that reflects the timing of the emergence (single-copy genes) or timing of duplication (multi-copy genes) of each gene, which we refer to as gene age. The inherent limitation with this approach is the availability of accurately annotated genome sequences across the phylogenetic tree. In the future, more phylogenetic information and additional gene matching algorithms will improve the fidelity of gene age prediction and may lead to some refining of the gene age categorization. Gene sorting examples are shown in Supplementary Fig. 2A and the complete list of genes can be found in Supplementary Data 4.

**Fig. 2:**
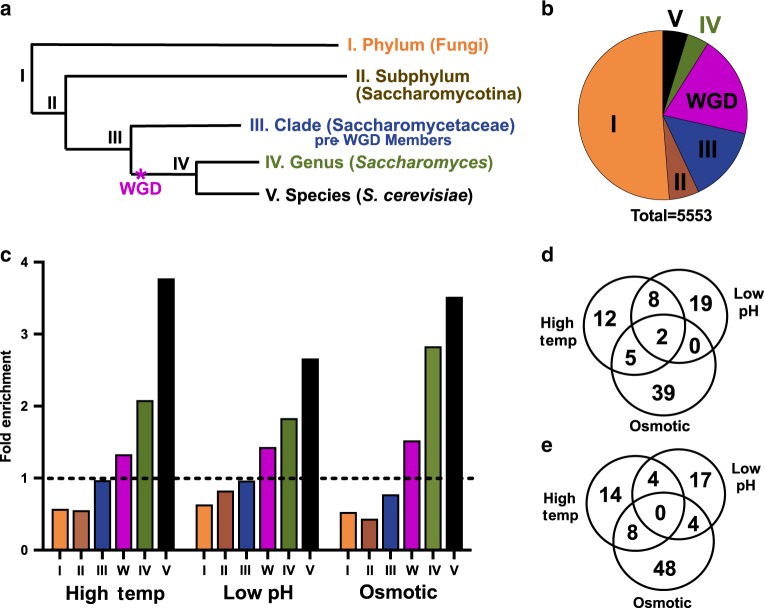
Stress adaptation responsive genes in *S. cerevisiae* are enriched for young genes. **a** A simplified phylogenetic tree for *S. cerevisiae* showing speciation events and the Whole Genome Duplication (magenta*). **b** The transcripts detected via RNAseq from this study were grouped based on ortholog presence in the groups shown (described in detail in Supplementary Fig. 4). **c** Differentially expressed genes for *S. cerevisiae* were parsed by their grouping shown in **b**, then normalized to the group size and the proportion of total Differentially Expressed (DE) genes per condition (dashed line). Transcripts in groups IV and V were assessed for shared upregulated genes (D) or downregulated genes (E).

The gene groupings in Fig. 2b were compared to the stress RNAseq data to determine the percentage of significantly differentially expressed genes in each age group. This analysis found a stepwise increase in the relative amount of differentially expressed genes in progressively younger gene groups in *S. cerevisiae*. Genes that were found to be conserved to filamentous fungi (ancient genes from group I) were 4.2 to 6.6-fold less likely to be differentially expressed after stress adaptation compared to *S. cerevisiae-*specific genes (group V) (Fig. 2c). Similar trends were observed when considering only upregulated or downregulated genes, however, upregulated genes showed a more pronounced bias towards young genes with 6.6 to 16.8-fold enrichment between group I and group V genes (Supplementary Fig. 5). Analysis of the expression pattern of young genes (those in groups IV and V) showed that few genes exhibited significantly changed expression in response to all stresses (Fig. 2d, e).

The findings in Fig. 2 were further tested by analyzing additional stress adaptation experiments for *S. cerevisiae* exposed to ethanol in a previous study^28^ or anaerobic stress (this study) (Supplementary Fig. 6). In both cases, young genes were enriched, and ancient genes were depleted amongst differentially expressed genes in response to stress adaptation. A similar enrichment for young genes was observed amongst varying amounts of ethanol stress, despite a difference in the number of total significant gene expression changes (Supplementary Fig. 6D). Together, these observations suggest that the sorting algorithm presented in Supplementary Fig. 4 is able to consistently identify a relationship between gene age and stress gene expression for several types and levels of stress in *S. cerevisiae*.

### Shared gene enrichment pattern across the Saccharomycotina

The findings in Fig. 2 showed an inverse correlation between gene age and stress differential expression in *S. cerevisiae*. If these findings were shared amongst other yeast species, they might imply an underlying evolutionary mechanism that can predict the genes that are more likely to be involved in stress adaptation. To test for a relationship between differential expression and gene age, we stratified the protein-coding genes of *K. marxianus* and *Y. lipolytica* using the same sorting concept described above for *S. cerevisiae* (Supplementary Fig. 4). The only modification to these sorting approaches was the elimination of the whole-genome duplication group, as neither of these species has undergone a recent whole-genome duplication^29,30^.

**Fig. 3:**
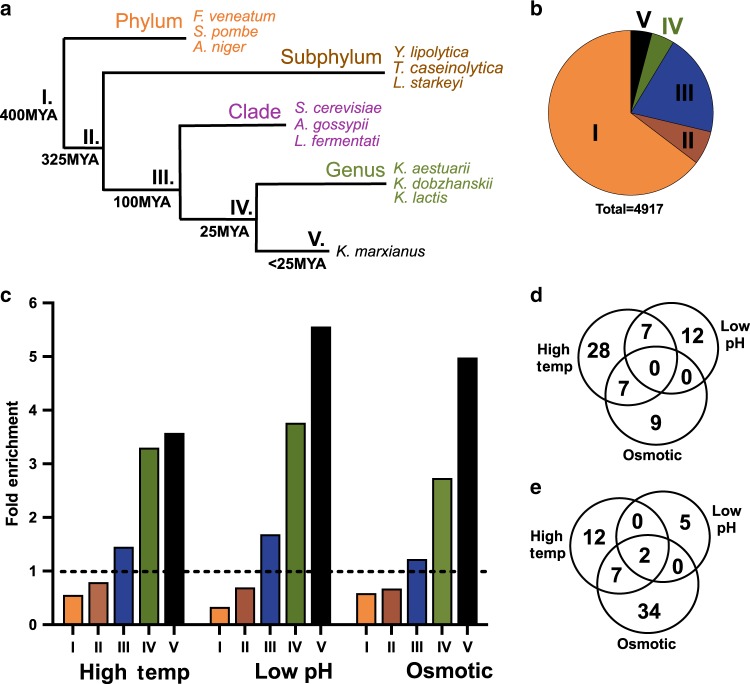
Stress adaptation responsive genes in *K. marxianus* are enriched for young genes. **a** A simplified phylogenetic tree for *K. marxianus* showing speciation events and organisms used in orthology queries. **b** The transcripts detected via RNAseq from this study were grouped based on ortholog presence in the groups shown (described in detail in Supplementary Fig. 4). **c** Differentially expressed genes for *K. marxianus* were parsed by their grouping shown in **a** and **b**, then normalized to the group size and the total measured DE % (dashed line). Transcripts in groups IV and V were assessed for shared upregulated genes (**d**) or downregulated genes (**e**).

Analysis of *K. marxianus* and *Y. lipolytica* gene groups in relation to each stress condition showed similar patterns to *S. cerevisiae*, with ancient genes exhibiting under-representation for significant differential expression compared to young gene groups (Fig. 3 and Supplementary Fig. 7). Also, as with *S*. cerevisiae, there were few young differentially expressed genes that responded to all stresses, suggesting that these expression changes were often condition specific (Fig. 3d, e). These biases towards young genes might explain the low observed overlap between significant expression changes amongst 1:1:1 orthologs shared between the three budding yeasts when exposed to the same type of stress (Supplementary Fig. 8). Together, these findings showed that in all three yeasts studied, young genes were enriched for long-term stress-responsiveness, or adaptation, compared to ancient genes. Further, since the species chosen for this analysis span much of the diversity of the budding yeast subphylum^21^, these results may be indicative of a shared stress adaptation mechanism, rather than a shared response of specific genes, amongst budding yeasts.

### Features of young genes are consistent with adaptive roles

**Fig. 4:**
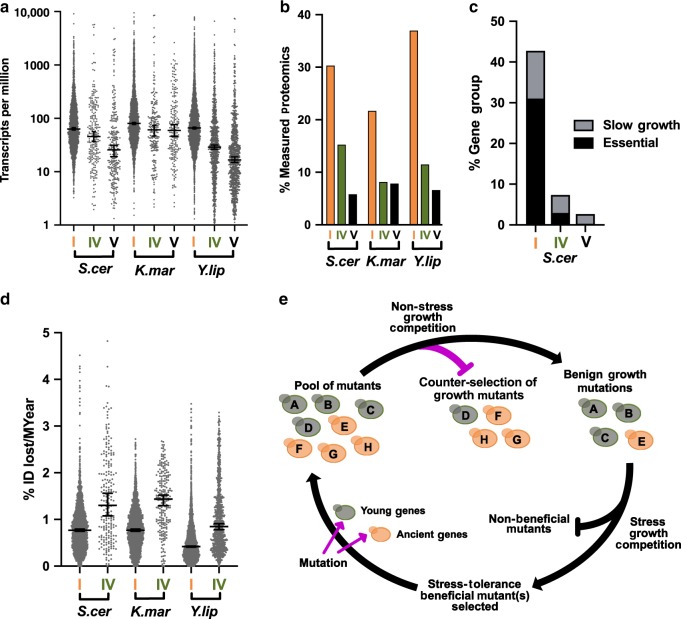
Less expressed and often non-essential young genes adapt more rapidly than ancient genes. **a** Standard growth condition RNAseq reads were normalized to the read depth and gene length to generate Transcripts per Million (TPM). Error bars at the 95% confidence interval of the median. **b** The percentage of mRNAs measured compared to proteins measured via mass spectrometry by quantifying eXtracted Ion Chromatograms. **c** The percentage of essential genes (black) and non-essential genes associated with slow growth (gray) is shown for *S. cerevisiae* ancient genes (I) and young genes (IV and V). Essential and slow growth ORFs were obtained from Giaever 2002^20^. **d** The percentage of amino acid identity changes for each protein in comparison to its closest homolog from a member of the same genus. Results were adjusted to the percent amino acid change per million years (% Intentity (ID) lost/MYear) using the estimated divergence time between pairs of organisms^13^. The median and 95% confidence interval is shown. Queries were performed between *S. cerevisiae/S. eubayanus*, *K. marxianus/K. lactis*, or *Y. lipolytica/Y. bubula*. E. A model for evolution to intermittent stress where random mutations occur amongst all genes (magenta arrows) followed by non-stress selection for benign mutants (magenta blocked arrow). Mutants that do not influence growth are selected upon stress exposure for fitness benefits. Source data underlying Fig. 4a, c, and d are provided as a source data file.

To understand the functions associated with the gene groupings produced in this study, we assessed biological processes associated with the ancient and young gene sets in *S. cerevisiae*, where ample functional information is available. This analysis showed ancient genes associated with fundamental biological processes including primary metabolism, tRNA aminoacylation, and DNA strand elongation, and 94% of these genes were annotated with at least one biological process GO term. Conversely, young genes (groups IV and V) were associated with more specialized functions like maltose transport, vitamin biosynthesis, and aldehyde metabolism, with many young genes lacking any biological process annotations in *S. cerevisiae* (40%). *K. marxianus* and *Y. lipolytica* also exhibited high percentages of young genes that were not associated with a biological process (41% and 69%, respectively) (Supplementary Fig. 9B). The fundamental nature of ancient gene functional associations was reflected by their high likelihood of being essential or required for optimal growth compared to young genes. Conversely, the more specialized functions of young genes were reflected by the 16-fold decrease in likelihood of growth impairment upon deletion compared to ancient genes (Fig. 4c)^31^. Analysis of cellular component enrichment showed that young proteins (groups IV and V) were significantly enriched for localization to the plasma membrane, cell wall, and vacuole, which was distinct from ancient proteins (group I) enrichment for nuclear, cytoplasmic, and mitochondrial localization (Supplementary Fig. 9B).

Further characterization of young protein-coding genes found that they exhibited lower median gene expression and their corresponding proteins were less frequently detected via mass spectrometry in non-stress samples compared to ancient genes (Figs. 4a, b). Previous works have shown that low expression and non-essentiality correlate with increased adaptation rates^32,33^, suggesting that young genes could adapt more rapidly compared to ancient genes. To test this, amino acid sequence identity was compared between homologous proteins from members of the same genus using BLAST+. Analysis of each protein sequence from groups I and IV allowed sequence identity changes to be compared over the same span of evolutionary time to assess adaptation rates. This analysis was adjusted to reflect the estimated evolutionary time elapsed^21^ between each pair of species and showed that the average frequency of amino acid identity changes was higher for young protein groups compared to ancient protein groups (Fig. 4d).

## Discussion

Budding yeasts are attractive for industrial production of biomolecules, since they grow rapidly, utilize inexpensive substrates, and are readily engineered to produce heterologous gene products^1–3^. However, stresses that result from feedstock composition, toxic products, and fluctuating reaction temperatures can lower the cost-effectiveness of industrial processes by diminishing productivity and yields^11^. Previous works have phenotypically characterized yeasts exhibiting stress tolerant phenotypes^22^, and whole genome sequencing data are available, but the genes that have evolved in these yeasts to enable survival and growth under unfavorable, stress-inducing conditions remain unclear. We now identify an association between stress-induced gene expression and gene age. We show that younger genes, namely, those that are restricted to a genus or species, or have duplicated in recent evolutionary time, are more likely to respond to different types of long-term stress, such as those that were imposed in continuous (chemostat) cultivation in this report. These stress-responsive genes can also be considered adaptation or niche-specialization genes as they have evolved to enable the yeasts carrying them tolerate ongoing harsh conditions.

The findings that adaptation rates and stress gene expression are biased toward young genes for three distantly related yeast species suggests an underlying evolutionary mechanism. The model in Fig. 4e suggests that during non-stress periods, ancient and young gene mutations may occur at similar rates, however, ancient genes may be subject to more stringent counter-selection (magenta blocked arrow) due to their higher expression and influence on growth (Fig. 4a, c). Conversely, non-synonymous mutations amongst young genes might accumulate more rapidly because these genes are rarely growth-related (Fig. 4c, d). The resulting increase in sequence space that is sampled by young genes would increase the probability of young mutants to enter stress-growth competition, thus increasing the chances of selecting young gene adaptations to benefit stress tolerance. We suggest that these events occur in a cyclical manner, enabling stress-tolerance functionalization of young genes without diminishing growth potential. This model could also apply to promoter sequences, which would enable specialized genes to adapt dynamic expression patterns in order to save resources during non-stress growth. This mechanism would explain the higher propensity of young genes to change expression in response to stress. The model might also provide an insight as to why improved stress tolerance in some laboratory-evolved strains comes at a cost to growth under standard growth conditions^34,35^. In this case, the relatively short, non-cyclical stresses applied during adaptive laboratory evolution does not allow for the counterselection of growth mutations.

In this work we found that young genes represented 4%, 5%, and 14% of protein-coding genes in *K. marxianus, S. cerevisiae*, and *Y. lipolytica*, respectively, which is in the same range as the 7-19% of genes in *C. elegans, D. melanogaster*, and *H. sapiens* that lack recognizable homologs in other organisms^26,36^. Previous works have linked some young genes to species and genus-specific adaptations, including movement on the surface of fast water in *Rhagovelia* water striders^37^, HIV-1 resistance in owl monkeys^38,39^, and the concurrent evolution of antifreeze proteins in several species^40–42^. Antifreeze protein genes are well-studied examples of young genes that arose via de novo gene origin events between 13 and 18 million years ago in codfishes and are present at variable copy number in some species^43^. Concurrently, the psychrophilic yeast *G. antarctica*, has evolved to encode nine antifreeze protein genes whose expression levels are induced by exposure to cold^15,44^. These attributes of antifreeze protein genes are similar to the young genes in this study, which were stress responsive, emerged in recent evolutionary time, and often exist at variable copy number. It seems plausible that the young, stress responsive genes described for *K. marxianus* could influence the capacity of this species to grow at higher temperatures (45 °C)^9^ than other members of the *Kluyveromyces* genus, like *K. lactis* (37 °C)^45^. Furthermore, the acquisition of this thermotolerant phenotype in a short span of evolutionary time would be consistent with the involvement of rapidly adapting young genes.

This study and previous stress tolerance investigations have identified dozens to hundreds of significant gene expression changes after stress exposure in budding yeasts^13,16–18,28^. Despite analysis of such stress-responsive genes in multiple species, rational engineering to further enhance robustness of industrial yeast strains remains difficult. The findings of this work suggest that considering the collective role of evolutionarily young stress-responsive genes from stress tolerant species is a pragmatic path forward towards achieving this goal. This suggestion is based on two points; first, single gene perturbations often fail to reproduce stress-response phenotypes^13^; and second, many mutations that improve stress tolerance cause trade-off phenotypes^10,34,35^. Establishing more robust industrial production strains may require modification of multiple genes and/or expression of several exogenous genes, while avoiding growth or physiological perturbations. To accomplish this, knowledge-driven approaches are needed to aid the identification of relevant genes that can be manipulated to confer the desired trait without negative consequences on growth. This goal is complicated by incomplete gene function information, especially for many stress tolerant yeast species. In this work, we present a gene sorting method that identifies a class of genes that are likely to be enriched in response to diverse stresses. By leveraging gene age information, it will be possible to focus rational experimental designs on unpredicted stress tolerance genes, which prior to this work fall into the category of genes of unknown function. Identifying these genes using this analysis methodology offers biotechnological potential as well as the tools to understand the process of species diversification and niche adaptation in yeast.

## Methods

### Strains and cultivation conditions

*Y. lipolytica* (W29), *K. marxianus* (CBS6556), and *S. cerevisiae* (CEN.PK113-7D) were grown in 30 mL synthetic media at 30 °C for 24 h in shake flasks, followed by inoculation of bioreactors and an initial batch growth phase. After the completion of the batch phase, chemostat cultivation was started with a dilution rate of 0.1/h and a working volume of 500 mL (*S. cerevisiae*) or 1 L (*K. marxianus* and *Y. lipolytica*). Stress conditions were achieved by altering either temperature, pH, or osmotic pressure (KCl) for the duration of the cultivation, specific conditions are listed in Supplementary Fig. 8. Standard growth temperature was adjusted to reflect organism specific tolerances. Cultivations for were performed in synthetic medium (SM)^46^ containing 5 g L^−1^ (NH_4_)_2_SO_4_, 3 g L^−1^ KH_2_PO_4_, 0.5 g L^−1^ MgSO_4_·7H_2_O, 7.5 g L^−1^ glucose, trace elements and vitamins with 1 g L^−1^ pluronic PE6100 to reduce foaming. Sample collection was carried out after at least five volume changes (50 h) in steady state growth conditions. At least three biological replicate experiments were performed for each species and each condition in this work. Steady state growth was defined as less than 5% deviation in biomass dry weight.

### Ortholog prediction with OrthoFinder

For Fig. 1, proteome-wide homology matching was executed using OrthoFinder^24^. Proteins were excluded from the core genome (non-core) if orthology search predicted zero orthologous proteins in any of the query species. Proteins were designated single-core if they were encoded by single-copy genes in the species (e.g., *S. cerevisiae HIS1*) or multi-core if they were duplicated in the species (e.g., *S. cerevisiae GAL1* and *GAL3*) (Supplementary Fig. 2). Protein groups were matched to their underlying genes for gene expression analyses. This grouping strategy was carried out to sort each species protein-coding genes into a single group. Results of these gene sorting analyses are shown in Supplementary Data 1, Supplementary Data 2 and Supplementary Data 3. For Figs. 2 and 3, and Supplementary Fig. 7, OrthoFinder was used to identify orthologs between each yeast and a set of eukaryotic organisms. This is shown in Supplementary Fig. 4 and is discussed in more detail in Supplementary Method 1. The results of these gene sorting analyses are shown in Supplementary Data 4, Supplementary Data 5 and Supplementary Data 6.

### RNAseq preparation and mapping

RNA extractions were performed on samples that were mechanically lysed with 0.5 mm acid-washed beads using an MP-Biomedicals FastPrep-24 for three one-minute cycles. Further extraction was performed using an RNeasy Kit from Qiagen. Libraries were prepared using the TruSeq mRNA Stranded HT kit. Sequencing was carried out using an Illumina NextSeq 500 High Output Kit v2 (75 bases), with a minimum of 8 million paired-end reads per replicate. The Novo Nordisk Foundation Centre for Biosustainability (Technical University of Denmark), performed the RNA sequencing and library preparation. RNAseq read mapping was performed after analysis in FASTQC, which identified one sample from *K. marxianus* as having overrepresented sequences. This sample was excluded from the analysis herein. Analysis for TPM in Fig. 4a was performed using Hisat2 v2.1.0^47^ and StringTie v1.3.3b^48^. RNAseq mapping for differential expression was mapped with STAR v2.7.0^49^ and reads were assigned with featureCounts v1.6.0^50^. Differential expression results can be found in Supplementary Data 1, Supplementary Data 2 and Supplementary Data 3.

### Differential expression analysis

Differential expression results were generated using limma v3.40.6^51^ and edgeR v3.26.8^52^ R packages and tidyverse v1.3.0^53^ was employed for various data rearrangements. Filtering was used to remove lowly expressed genes/proteins, and each dataset was filtered to remove genes/proteins for which the relative standard deviation was greater than 1 (RSD > 1) across replicates for a given condition and organism. Differential expression was defined by a significance cutoff of absolute log_2_FC > 1 and False Discovery Rate < 0.01 for a stress condition compared to control. The data analysis pipeline is described in Supplementary Method 6.

### Reporting Summary

Further information on research design is available in the Nature Research Reporting Summary linked to this article.

## Supplementary information

Supplementary Information

Peer Review File

Reporting Summary

Description of Additional Supplementary Files

Supplementary Data 1-6

## Data Availability

Data supporting the findings of this work are available within the paper and its Supplementary Information files. A reporting summary for this Article is available as a Supplementary Information file. All mapped transcript data and protein detection data generated in this work can be found at https://github.com/SysBioChalmers/OrthOmics. RNAseq datasets of data generated in this study can be found using SRA accession PRJNA531619 [https://www.ncbi.nlm.nih.gov/bioproject/PRJNA531619/]. Additional RNAseq data analyzed in Supplementary Fig. 6 are available in the ArrayExpress database with the dataset ID E-MTAB-4044 [https://www.ebi.ac.uk/arrayexpress/experiments/E-MTAB-4044/]. Proteomics data is available via the PRIDE partner repository with the dataset ID PXD011426 [http://proteomecentral.proteomexchange.org/cgi/GetDataset?ID=PXD011426]. The source data underlying Figs. 4a, c, and d, as well as Supplementary Figs. 1A, 2B, 3B, 3C, 6B, 6D, and 9 are provided as a Source Data file.

## Source Data

Source Data

## Code availability

All custom tools and analysis scripts can be freely accessed at github repository [https://github.com/SysBioChalmers/OrthOmics].

## References

[1] Mohd Azhar SH (2017). Yeasts in sustainable bioethanol production: a review. Biochem. Biophys. Rep..

[2] Nielsen J, Keasling JD (2016). Engineering cellular metabolism. Cell.

[3] Sanchez-Garcia L (2016). Recombinant pharmaceuticals from microbial cells: a 2015 update. Microb. Cell Fact..

[4] Ma YJ, Lin LL, Chien HR, Hsu WH (2000). Efficient utilization of starch by a recombinant strain of *Saccharomyces cerevisiae* producing glucoamylase and isoamylase. Biotechnol. Appl. Biochem..

[5] Ledesma-Amaro R, Nicaud JM (2016). *Yarrowia lipolytica* as a biotechnological chassis to produce usual and unusual fatty acids. Prog. Lipid Res..

[6] Varela, J. A., Gethins, L., Stanton, C., Ross, P. & Morrissey, J. P. Applications of *Kluyveromyces marxianus* in biotechnology. In *Yeast Diversity in Human Welfare* (eds. Satyanarayana, T. & Kunze, G.) 439–453 (Springer, Singapore, 2017). 10.1007/978-981-10-2621-8_17

[7] Nicaud J-M (2012). Yarrowia lipolytica. Yeast.

[8] Gonçalves FAG, Colen G, Takahashi JA (2014). *Yarrowia lipolytica* and its multiple applications in the biotechnological industry. Sci. World J..

[9] Lane MM, Morrissey JP (2010). *Kluyveromyces marxianus*: a yeast emerging from its sister’s shadow. Fungal Biol. Rev..

[10] Mans R, Daran JG, Pronk JT (2018). Under pressure: evolutionary engineering of yeast strains for improved performance in fuels and chemicals production. Curr. Opin. Biotechnol..

[11] Deparis, Q., Claes, A., Foulquie-Moreno, M. R. & Thevelein, J. M. Engineering tolerance to industrially relevant stress factors in yeast cell factories. *FEMS Yeast Res*. **17**, 10.1093/femsyr/fox036 (2017). 10.1093/femsyr/fox036 PMC5812522 28586408

[12] Caspeta Y (2014). Altered sterol composition renders yeast thermotolerant. Science.

[13] Gibney PA, Lu C, Caudy AA, Hess DC, Botstein D (2013). Yeast metabolic and signaling genes are required for heat-shock survival and have little overlap with the heat-induced genes. Proc. Natl. Acad. Sci. USA.

[14] Lahtvee P-J, Kumar R, Hallström BM, Nielsen J (2016). Adaptation to different types of stress converge on mitochondrial metabolism. Mol. Biol. Cell.

[15] Firdaus-Raih M (2018). The *Glaciozyma antarctica* genome reveals an array of systems that provide sustained responses towards temperature variations in a persistently cold habitat. PLoS ONE.

[16] Silva A (2017). Regulation of transcription elongation in response to osmostress. PLoS Genet..

[17] Hughes Hallett JE, Luo X, Capaldi AP (2014). State transitions in the TORC1 signaling pathway and information processing in *Saccharomyces cerevisiae*. Genetics.

[18] Kasavi C, Eraslan S, Oner ET, Kirdar B (2016). An integrative analysis of transcriptomic response of ethanol tolerant strains to ethanol in *Saccharomyces cerevisiae*. Mol. Biosyst..

[19] Botstein D, Fink GR (2011). Yeast: an experimental organism for 21st Century biology. Genetics.

[20] Conesa A (2005). Blast2GO: a universal tool for annotation, visualization and analysis in functional genomics research. Bioinformatics.

[21] Shen XX (2018). Tempo and mode of genome evolution in the budding yeast subphylum. Cell.

[22] Buzzini P, Turchetti B, Yurkov A (2018). Extremophilic yeasts: the toughest yeasts around?. Yeast.

[23] O’Duibhir E (2014). Cell cycle population effects in perturbation studies. Mol. Syst. Biol..

[24] Emms DM, Kelly S (2015). OrthoFinder: solving fundamental biases in whole genome comparisons dramatically improves orthogroup inference accuracy. Genome Biol..

[25] Prachumwat A, Li W-H (2005). Protein function, connectivity, and duplicability in yeast. Mol. Biol. Evol..

[26] Domazet-Lošo T, Brajković J, Tautz D (2007). A phylostratigraphy approach to uncover the genomic history of major adaptations in metazoan lineages. Trends Genet..

[27] Byrne KP, Wolfe KH (2005). The yeast gene order browser: combining curated homology and syntenic context reveals gene fate in polyploid species. Genome Res..

[28] Lahtvee P-J (2017). Absolute quantification of protein and mRNA abundances demonstrate variability in gene-specific translation efficiency in yeast. Cell Syst..

[29] Wolfe KH (2015). Origin of the yeast whole-genome duplication. PLoS Biol..

[30] Magnan C (2016). Sequence assembly of *Yarrowia lipolytica* strain W29/CLIB89 shows transposable element diversity. PLoS ONE.

[31] Giaever G (2002). Functional profiling of the *Saccharomyces cerevisiae* genome. Nature.

[32] Pál C, Papp B, Hurst LD (2001). Highly expressed genes in yeast evolve slowly. Genetics.

[33] Mata Bahler JJ (2003). Correlations between gene expression and gene conservation in fission yeast. Genome Res..

[34] Huang C-J, Lu M-Y, Chang Y-W, Li W-H (2018). Experimental evolution of yeast for high-temperature tolerance. Mol. Biol. Evol..

[35] Caspeta L, Chen Y, Nielsen J (2016). Thermotolerant yeasts selected by adaptive evolution express heat stress response at 30 °C. Sci. Rep..

[36] Khalturin K, Hemmrich G, Fraune S, Augustin R, Bosch TC (2009). More than just orphans: are taxonomically-restricted genes important in evolution?. Trends Genet..

[37] Santos ME, Le Bouquin A, Crumière AJJ, Khila A (2017). Taxon-restricted genes at the origin of a novel trait allowing access to a new environment. Science.

[38] Sayah DM, Sokolskaja E, Berthoux L, Luban J (2004). Cyclophilin A retrotransposition into TRIM5 explains owl monkey resistance to HIV-1. Nature.

[39] Stremlau M (2004). The cytoplasmic body component TRIM5alpha restricts HIV-1 infection in Old World monkeys. Nature.

[40] Zhuang X, Yang C, Murphy KR, Cheng C-HC (2019). Molecular mechanism and history of non-sense to sense evolution of antifreeze glycoprotein gene in northern gadids. Proc. Natl. Acad. Sci. USA.

[41] Chen L, DeVries AL, Cheng CH (1997). Convergent evolution of antifreeze glycoproteins in Antarctic notothenioid fish and Arctic cod. Proc. Natl. Acad. Sci. USA.

[42] Chen S, Krinsky BH, Long M (2013). New genes as drivers of phenotypic evolution. Nat. Rev. Genet..

[43] Baalsrud HT (2017). De novo gene evolution of antifreeze glycoproteins in codfishes revealed by whole genome sequence data. Mol. Biol. Evol..

[44] Hashim NHF (2013). Characterization of Afp1, an antifreeze protein from the psychrophilic yeast Glaciozymaantarctica PI12. Extremophiles.

[45] Steensma HYM, de JFC, Linnekamp M (1988). The use of electrophoretic karyotypes in the classification of yeasts: *Kluyveromyces marxianus* and *K. lactis*. Curr. Genet..

[46] Verduyn C, Postma E, Scheffers WA, Van Dijken JP (1992). Effect of benzoic acid on metabolic fluxes in yeasts: a continuous‐culture study on the regulation of respiration and alcoholic fermentation. Yeast.

[47] Kim D, Langmead B, Salzberg SL (2015). HISAT: a fast spliced aligner with low memory requirements. Nat. Methods.

[48] Pertea M (2015). StringTie enables improved reconstruction of a transcriptome from RNA-seq reads. Nat. Biotechnol..

[49] Dobin A (2013). STAR: ultrafast universal RNA-seq aligner. Bioinformatics.

[50] Liao Y, Smyth GK, Shi W (2014). featureCounts: an efficient general purpose program for assigning sequence reads to genomic features. Bioinformatics.

[51] Ritchie ME (2015). *limma* powers differential expression analyses for RNA-sequencing and microarray studies. Nucleic Acids Res..

[52] McCarthy DJ, Chen Y, Smyth GK (2012). Differential expression analysis of multifactor RNA-Seq experiments with respect to biological variation. Nucleic Acids Res..

[53] Wickham H (2019). Welcome to Tidyverse. J. Open Source Softw..

