# Supplementary material for: Stress-induced expression is enriched for evolutionarily young genes in diverse budding yeasts
Source: Nat Commun. 2020 May 1;11:2144. doi: 10.1038/s41467-020-16073-3 (PMC7195364; doi:10.1038/s41467-020-16073-3)
Supplement: Supplementary file 3 — Reporting Summary [file 41467_2020_16073_MOESM3_ESM.pdf]

nature research

Corresponding author(s): John P Morrissey

Last updated by author(s): Apr 7, 2020

Reporting Summary

Nature Research wishes to improve the reproducibility of the work that we publish. This form provides structure for consistency and transparency in reporting. For further information on Nature Research policies, see Authors & Referees and the Editorial Policy Checklist .

Statistics

For all statistical analyses, confirm that the following items are present in the figure legend, table legend, main text, or Methods section.

n/a	Confirmed	
☐	☒	The exact sample size (n) for each experimental group/condition, given as a discrete number and unit of measurement
☐	☒	A statement on whether measurements were taken from distinct samples or whether the same sample was measured repeatedly
☐	☒	The statistical test(s) used AND whether they are one- or two-sided<br>*Only common tests should be described solely by name; describe more complex techniques in the Methods section.*
☐	☒	A description of all covariates tested
☐	☒	A description of any assumptions or corrections, such as tests of normality and adjustment for multiple comparisons
☐	☒	A full description of the statistical parameters including central tendency (e.g. means) or other basic estimates (e.g. regression coefficient) AND variation (e.g. standard deviation) or associated estimates of uncertainty (e.g. confidence intervals)
☐	☒	For null hypothesis testing, the test statistic (e.g. F, t, r) with confidence intervals, effect sizes, degrees of freedom and P value noted<br>*Give P values as exact values whenever suitable.*
☒	☐	For Bayesian analysis, information on the choice of priors and Markov chain Monte Carlo settings
☒	☐	For hierarchical and complex designs, identification of the appropriate level for tests and full reporting of outcomes
☒	☐	Estimates of effect sizes (e.g. Cohen's d, Pearson's r), indicating how they were calculated

Our web collection on statistics for biologists contains articles on many of the points above.

Software and code

Policy information about availability of computer code

Data collection	Blast2GO 5 Pro was used to assess which genes in K. marxianus and Y. lipolytica lacked Biological Process annotations
Data analysis	OrthoFinder v 2.3.3 was used to identify ortholog groups used in this study.<br>FastQC 0.11.8 was used to assess RNAseq sample quality<br>STAR 2. 7.0 and featurecounts vl.6.0 were used for to map RNAseq for differential expression<br>X!Tandem algorithm version Alanine 2017.02.01 and X!TandemPipeline version 3.4.4 were used to identify proteins<br>R-packages used for differential expression analysis - edgeR 3.26.8, limma 3.40.6, and tidyverse 1.3.0<br>String Tie vl.3.3b and HISAT2 v2.l.0 were used to determine TPM for RNAseq data.<br>All custom tools and analysis scripts can be freely accessed at a github repository [https://github.com/SysBioChalmers/OrthOmics].

For manuscripts utilizing custom algorithms or software that are central to the research but not yet described in published literature, software must be made available to editors/reviewers. We strongly encourage code deposition in a community repository (e.g. GitHub). See the Nature Research guidelines for submitting code & software for further information.

Data

Policy information about availability of data

All manuscripts must include a data availability statement. This statement should provide the following information, where applicable:

- Accession codes, unique identifiers, or web links for publicly available datasets
- A list of figures that have associated raw data
- A description of any restrictions on data availability

- Data supporting the findings of this work are available within the paper and its Supplementary Information files.
- A reporting summary for this Article is available as a Supplementary Information file.
- All mapped transcript data and protein detection data generated in this work can be found at https://github.com/SysBioChalmers/OrthOmics. RNAseq datasets of data generated in this study can be found using SRA accession PRJNA531619 [https://www.ncbi.nlm.nih.gov/sra/?term=PRJNA531619. Additional RNAseq data

analyzed in Supplementary Fig. 6 are available in the ArrayExpress database with the dataset ID E-MTAB-4044 [https://www.ebi.ac.uk/arrayexpress/experiments/E-MTAB-4044/]. Proteomics data is available via the PRIDE partner repository with the dataset ID PXD011426 [http://proteomecentral.proteomexchange.org/cgi/GetDataset?ID=PXD011426].
- Supplementary data files provide the source data underlying Fig. 1 (Supplementary Data 1-3); Fig. 2 (Supplementary Data 4); Fig. 3 (Supplementary Data 5); Supplementary Fig 5. (Supplementary Data 4); Supplementary Fig. 7 (Supplementary Data 6); and Supplementary Fig. 8 (Supplementary Data 1 -3). The source data underlying Fig. 4, Supplementary Fig. 1, Supplementary Fig. 2, Supplementary Fig. 3, Supplementary Fig. 6 and Supplementary Fig. 9 are provided as a Source Data file.

Field-specific reporting

Please select the one below that is the best fit for your research. If you are not sure, read the appropriate sections before making your selection.

☒ Life sciences ☐ Behavioural & social sciences ☐ Ecological, evolutionary & environmental sciences

For a reference copy of the document with all sections, see nature.com/documents/nr-reporting-summary-flat.pdf

Life sciences study design

All studies must disclose on these points even when the disclosure is negative.

Sample size	No calculation was performed to determine sample size. Sample sizes were chosen to cover the maximum number of species and stress conditions while maintaining replicates to inform statistical analyses.
Data exclusions	One RNAseq library from the K. marxianus high temperature chemostats was excluded from further analysis due to the presence of low library quality and highly overrepresented sequences when analyzed with the bioinformatic tool FastQC. This phenomenon was unique to this sample. The intention to exclude samples that failed FastQC was pre-determined prior to the analysis.
Replication	Each experimental condition included three biological replicates in a separate fermenter. For omics data, genes or proteins that were measured were excluded if the relative standard deviation between replicate samples was greater than 1. All attempts at replication were successful.
Randomization	Samples were grouped first by species, then by the growth conditions established prior to sampling. Covariates were controlled by establishing steady state growth conditions in continuously fed chemostats for 50 hours prior to sample collection.
Blinding	Blinding was not employed for this study as it was necessary to know the species from which samples were obtained in order to perform the bioinformatics.

Reporting for specific materials, systems and methods

We require information from authors about some types of materials, experimental systems and methods used in many studies. Here, indicate whether each material, system or method listed is relevant to your study. If you are not sure if a list item applies to your research, read the appropriate section before selecting a response.

Materials & experimental systems

n/a	Involved in the study
☒	☐ Antibodies
☒	☐ Eukaryotic cell lines
☒	☐ Palaeontology
☒	☐ Animals and other organisms
☒	☐ Human research participants
☒	☐ Clinical data

Methods

n/a	Involved in the study
☒	☐ ChIP-seq
☒	☐ Flow cytometry
☒	☐ MRI-based neuroimaging
