# Supplementary material for: Stress-induced expression is enriched for evolutionarily young genes in diverse budding yeasts
Source: Nat Commun. 2020 May 1;11:2144. doi: 10.1038/s41467-020-16073-3 (PMC7195364; doi:10.1038/s41467-020-16073-3)
Supplement: Supplementary file 4 — Description of Additional Supplementary Files [file 41467_2020_16073_MOESM4_ESM.docx]

**Description of Additional Supplementary Files**

File name: Supplementary Data 1
Description: Protein-coding genes of *S. cerevisiae* sorted by inferred orthology categories across *S. cerevisiae*, *K. marxianus* and *Y. lipolytica*. Results for differential expression analysis of mRNAs are shown for conditions of high temperature, low pH and osmotic stress compared to reference.

File name: Supplementary Data 2
Description: Protein-coding genes of *K. marxianus* sorted by inferred orthology categories across *S. cerevisiae*, *K. marxianus* and *Y. lipolytica*. Results for differential expression analysis of mRNAs are shown for conditions of high temperature, low pH and osmotic stress compared to reference.

File name: Supplementary Data 3
Description: Protein-coding genes of *Y. lipolytica* sorted by inferred orthology categories across *S. cerevisiae*, *K. marxianus* and *Y. lipolytica*. Results for differential expression analysis of mRNAs are shown for conditions of high temperature and low pH compared to reference.

File name: Supplementary Data 4
Description: Protein-coding genes of *S. cerevisiae* sorted by gene age groups (Groups I, II, III, IV and V correspond to genes conserved at the phylum, subphylum, clade, genus and species level. WGD indicates genes from the whole genome duplication event). Results for differential expression analysis of mRNAs are shown for conditions of high temperature, low pH and osmotic stress compared to reference.

File name: Supplementary Data 5
Description: Protein-coding genes of *K. marxianus* sorted by gene age groups (Groups I, II, III, IV and V correspond to genes conserved at the phylum, subphylum, clade, genus and species level). Results for differential expression analysis of mRNAs are shown for conditions of high temperature, low pH and osmotic stress compared to reference.

File name: Supplementary Data 6
Description: Protein-coding genes of *Y. lipolytica* sorted by gene age groups (Groups I, II, III, IV and V correspond to genes conserved at the phylum, subphylum, clade, genus and species level). Results for differential expression analysis of mRNAs are shown for conditions of high temperature and low pH compared to reference.
