# Supplementary material for: Stress-induced expression is enriched for evolutionarily young genes in diverse budding yeasts
Source: Nat Commun. 2020 May 1;11:2144. doi: 10.1038/s41467-020-16073-3 (PMC7195364; doi:10.1038/s41467-020-16073-3)
Supplement: Supplementary file 2 — Peer Review File [file 41467_2020_16073_MOESM2_ESM.pdf]

Reviewers' comments:

Reviewer #1 (Remarks to the Author):

The authors present a very interesting paper on the importance of young vs ancient genes across three yeast hosts with respect to stress response. Overall, the analysis and conclusions associated with this paper are of interest and relevance to both biotechnology as well as basic molecular biology/genetics.

My major issue with the paper lies within a systematic flaw across the experimental set-up that should be addressed in some way (either as a major caveat on the work or through repeating some experimentation). Specifically, the authors fixed the dilution rate to 0.1 hr-1 in the chemostats and applied a constant stress condition as outlined in Supplemental information 1A. In general, the stresses were seemingly random as to the degree of stress imposed (this can be seen with the inability to get an osmotic stress conditions for Y. lipolytica). What would be more important is the magnitude of the stress compared to the basal level tolerance of the strain. For example, the forced growth rate of 0.1 hr-1 represents a different percentage of maximal growth for each strain. This should be discussed. The constant selection of a pH of 3.5 for the low pH condition is curious as this represents a differential pH tolerance for cells (with K marxianus and Y. lipolytica being more used to a lower pH). It would be more telling to (like with growth rate) see the percentage of minimum inhibitory concentration and have this be the threshold of the stress, not the magnitude of the external condition. It can be hypothesized that the degree of response in a cell will be proportional the amount of stress. Thus, if the degree of stress a cell is seeing is not the same across all cells, it is possible that this data analysis is comparing apples to oranges. This point needs to be better considered in the paper to gain confidence in the work.

Reviewer #2 (Remarks to the Author):

1 – What are the major claims of the paper? Are they novel and will they be of interest to others in the community and the wider field? If the conclusions are not original, it would be helpful if you could provide relevant references.
I like this paper and the idea of testing stress response in organisms that are relevant and in settings that sort of mimic what the organisms meet in biotechnological and industrial production settings.
The main claim of this paper is that the results show that "few significant changes were shared between [yeast] organisms after adaptation to the same stress. … [G]enes that were shared broadly amongst eukaryotes (ancient genes) were less responsive to stress than genus or species-specific (young) genes for each of the yeasts in response to each stress." The results support this analysis and it is consistent with previous results (see the references below for some examples).
A minor (to me) claim is that "This work presents a novel approach to understanding stress gene expression and finds that efforts to engineer stress tolerance could benefit from consideration of the evolutionary age and adaptation rate of target genes."
I am not entirely sure about the novelty of this approach. It is established that stress responsive genes often evolve by gene duplication followed by divergence. Example are analyzed in https://royalsocietypublishing.org/doi/10.1098/rsos.171198 or https://doi.org/10.1371/journal.pgen.1000581. This means that stress- responsive genes tend to be younger genes. This was also found in an analysis of yeast genome, where genes that change their expression during stress response are enriched in unknown function and depleted from genes involved in complexes (https://doi.org/10.1371/journal.pcbi.1000674). Further, comparative analyses of bacterial genomes suggest that evolution of stress response genes tends to be network-modular (to different degrees) and that innovation tends to center around the "sensor" genes rather than around "transmitter" or "actuator" genes (https://www.pnas.org/content/105/21/7500).
Thus, in my mind it is already established that "engineer[ing] stress tolerance could benefit from

consideration of the evolutionary age and adaptation rate of target genes."

2 - Is the work convincing, and if not, what further evidence would be required to strengthen the conclusions?
I have some methodological doubts about the work. While I realize that comparing 1:1:1 orthologs makes the analysis simpler, it also masks the nuances.
In addition, the methods should be more detailed, as in many cases I could not understand the specifics of how the analysis was done.
I remark that I am not doubting the analysis, I just think it should be explained in a way that one could reproduce it without having to guess the intermediate steps.
Also, by analyzing 1:1:1 orthologs you are working against yourselves. If you are correct, then stress response genes should be enriched in genes for which there are no orthologs in the other yeast or for which there has been duplication, and the duplicated genes could evolve and gains new biological function. I would add this analysis to you work.

3 – On a more subjective note, do you feel that the paper will influence thinking in the field? Please feel free to raise any further questions and concerns about the paper.

This is a question the journal should either define better or entirely remove from your reviewer instructions. I have grown old enough to realize that influence in the thinking of a field tend to be more tied to luck, networking and general politics than to a specific paper or set of results.
Hence, the editors, as gatekeepers are much influent in determining/predicting/setting this up than the reviewers.

4 – Specific comments and suggestions:
Page 3: "Other yeast species adaptations that make them well-suited for production of specific biomolecules, but have been difficult to engineer until the recent emergence of CRISPR/Cas95,6."
Unclear. Please rephrase and clarify what you mean.
Page 3: "Analysis of 2,903 orthologous protein coding genes found that few significant changes were shared between these organisms after adaptation to the same stress."
At this stage the reader questions why only analyzing 2 903 orthologous genes and not the entire genomes. Only latter the reason is given. I would rephrase this in order not to put the reader in a mindset "Why not6 look at them all?" from the beginning.
Page 4: "To assess the fidelity of ortholog predictions, protein complexes and enzymatic processes that are expected to be conserved amongst budding yeasts were searched for amongst the core proteins18."
Please describe how this comparison was done in detail in methods.

Page 5: "Core Genes and Proteins are Unenriched for Stress-Responsive Expression"
This should be "Core Genes and Proteins are depleted for Stress-Responsive Expression"

Page 5 "A separate gene grouping analysis was carried out for each yeast to include relatives that had similar estimated times of common ancestor divergence. Orthologous gene predictions were used to sort each protein, and its coding gene into a single bin based on the most distant ancestor with a predicted orthologous protein (Supplemental Figure 3a and Supplemental Figure 3b)."
Unclear what exactly was done here. Please expand and clearly explain the analysis, controls and comparisons.

Page 6: "Together, these findings show that the young genes were enriched for stress-responsiveness compared ancient genes for each yeast species in this study after adaptation to each stress condition."
Please correct sentence

Page 6: "As the species chosen for this analysis span much of the diversity of the budding yeast

subphylum13, these results may be indicative of a conserved stress adaptation mechanism amongst budding yeasts."

Sure, and the mechanism is likely to be gene/module duplication followed by divergent evolution, which is common to other organisms (see references above).

Page 6: "Stress shock and stress adaptation exhibit different gene expression changes (Supplemental Figure 1b and 1c), which may be indicative of distinct response mechanisms."
Supp. Figs 1b (especially) and 1c do not really allow me to see this. There is a large commonality between the two sets. If I am right, a plot taking all genes from the two sample being compared and plotting change-fold or (quantile of change-fold) should have a strong correlation/Adjusted R squared.

Page 6: "experiments from Hughes Hallett 2014 showed"

Please fix.

Page 6-7: "In contrast, the stress adaptation experiments from this report showed young genes were enriched amongst both upregulated and downregulated genes in response to osmotic and temperature stress (Supplemental 7c and 7f). These results suggest that stress shock and stress adaptation gene expression differ in relation to gene age, especially amongst downregulated genes."
I think the authors could propose possible interpretations for this observation. Results from https://doi.org/10.1371/journal.pcbi.1000674 and the reference you give show that early stress response is enriched in "young genes". If there is a second set/module of stress responsive genes responsible for long term adaptation, then it would be plausible that the early response module is down regulated (hence enrichment in younger genes in the set of down-regulated genes during long term response) and the later adaptation module is upregulated (hence enrichment in younger genes in the set of down-regulated genes during long term response). If this interpretation is correct, the set of downregulated young genes in long term response should have a large overlap with the set of young genes upregulated in the early response. This can be checked in your datasets.

Page 7: "Young Genes are Less Expressed, Shorter, Less Likely to be Essential, and Accumulate More Sequences Changes than Ancient Genes."
I don't think results in this section are entirely new. See the references above.

Page 10: Please correct typos
"media for 24hours in"; "listed inSupplemental 1A."; "Cultivations for were performed"

Page 10: "Steady state growth was defined as less than 5% deviation in biomass dry weight."
Please describe how this was assessed.

Page 11: "MS data are available online on public databases via the PRIDE39 repository with the dataset identifier PXD011426 and PROTICdb37,40 or via the following URLs:
http://moulon.inra.fr/protic/chassy_kluyveromyces
http://moulon.inra.fr/protic/chassy_yarrowia
http://moulon.inra.fr/protic/chassy_saccharomyces"
Please make sure these datasets are public. I tried to access them to make sure they were accessible and they were behind a password.

Reviewer #3 (Remarks to the Author):

The manuscript by Doughty et al describes their study of stress response in several budding yeasts

species: S. cerevisiae, K. marxianus and Y. lipolytica. They identify genes that are differentially expressed under various stress conditions in those taxa, and then look if those genes are conserved only at species/genus level (a.k.a. young genes) or at more broad evolutionary scale (ancient genes). This analysis shows that young genes are enriched in the cellular stress-response repertoire, and the authors propose that they might be high-value targets for improving industrial performance of yeast strains. Overall the manuscript is well-written and the chosen approach is appropriate for this type of investigation.

My main concern is with the limited scope of the evolutionary analysis used to determine which genes are species/genus specific, and thus can be categorized as 'young'. Given how much of the paper's impact and conclusions really hangs on this classification, simply using a single other species from a specific genus as well as further out (for the classifications into groups I-III) can potentially intriduce very serious bias. This omission is especially jarring since literally hundreds of budding yeast genomes and proteomes are currently publicly available. While I by no means suggest that the authors survey the entirety of Saccharomycotina, I think that increasing the depth and sampling of the evolutionary analysis across the subphylum is absolutely necessary for the authors' results and conclusions to be fully supported. Checking gene conservation among a few dozen (30-40) yeast taxa, well-spaced across the budding yeast lineage should suffice to get a more robust picture of which genes truly are young and which are ancient.

Other than this, I do not have any major or minor issues with the paper, except maybe for the use of the term "unenriched for", where I think "depleted for" would be more appropriate.

Response to the Reviewers' comments.

As indicated in our correspondence, in the context of an appeal for a re-consideration, we address all the issues raised in the original review. I put the comments in order of reviewer 1, reviewer 3 and reviewer 2 as the responses make more sense this way.

Reviewer #1 (Remarks to the Author):

The authors present a very interesting paper on the importance of young vs ancient genes across three yeast hosts with respect to stress response. Overall, the analysis and conclusions associated with this paper are of interest and relevance to both biotechnology as well as basic molecular biology/genetics.

My major issue with the paper lies within a systematic flaw across the experimental set-up that should be addressed in some way (either as a major caveat on the work or through repeating some experimentation). Specifically, the authors fixed the dilution rate to 0.1 hr-1 in the chemostats and applied a constant stress condition as outlined in Supplemental information 1A. In general, the stresses were seemingly random as to the degree of stress imposed (this can be seen with the inability to get an osmotic stress conditions for Y. lipolytica). What would be more important is the magnitude of the stress compared to the basal level tolerance of the strain. For example, the forced growth rate of 0.1 hr-1 represents a different percentage of maximal growth for each strain. This should be discussed. The constant selection of a pH of 3.5 for the low pH condition is curious as this represents a differential pH tolerance for cells (with K marxianus and Y. lipolytica being more used to a lower pH). It would be more telling to (like with growth rate) see the percentage of minimum inhibitory concentration and have this be the threshold of the stress, not the magnitude of the external condition. It can be hypothesized that the degree of response in a cell will be proportional the amount of stress. Thus, if the degree of stress a cell is seeing is not the same across all cells, it is possible that this data analysis is comparing apples to oranges. This point needs to be better considered in the paper to gain confidence in the work.

We agree with the fundamental point – that while we have applied the same stress to the different yeast species, the *degree* of stress may not be the same for each. The reviewer feels that the design of the experiment does not allow a fair comparison between the different species for each stress. In fact, as we analysed this comment, it became obvious that this was not a relevant comparison for us to be doing in any case. The important comparisons are *within each species* - in other words, what changes in gene expression happen in response to different stressful growth conditions in each species. In the revised manuscript, we do not compare particular genes *between species* but focus on whether there are common trends, so the issue raised by the reviewer is no longer a concern.

This goal of comparing within each species is explained in lines 108 – 112 in the revised manuscript. We do not try to find a conserved response because as indicated by the first results heading ("Lines 122-123 - Stress Adaptation Responsive Genes are Enriched for Duplicated and Non-Conserved Genes"), the enrichment we see is in non-conserved genes. This basic conclusion (but without the major additions in response to reviewer 3) was also in our first manuscript but was obscured when we first tried to find a conserved response that did not exist. Ultimately, the reviewers comment about differences in the magnitude of stress could be viewed as a strength of the conclusion, as each organism, stress type, and degree of stress showed enrichment for young genes. The effect of the degree of stress is assessed directly in our updated draft in Supplementary Figure 6D. In this figure we applied our gene grouping strategy to gene expression data collected from strains exposed to varying degrees of ethanol stress and found that in each case, expression responses were enriched for young genes.

Reviewer #3 (Remarks to the Author):

The manuscript by Doughty et al describes their study of stress response in several budding yeasts species: S. cerevisiae, K. marxianus and Y. lipolytica. They identify genes that are differentially expressed under various stress conditions in those taxa, and then look if those genes are conserved only at species/genus level (a.k.a. young genes) or at more broad evolutionary scale (ancient genes). This analysis shows that young genes are enriched in the cellular stress-response repertoire, and the authors propose that they might be high-value targets for improving industrial performance of yeast strains. Overall the manuscript is well-written and the chosen approach is appropriate for this type of investigation.

My main concern is with the limited scope of the evolutionary analysis used to determine which genes are species/genus specific, and thus can be categorized as 'young'. Given how much of the paper's impact and conclusions really hangs on this classification, simply using a single other species from a specific genus as well as further out (for the classifications into groups I-III) can potentially introduce very serious bias. This omission is especially jarring since literally hundreds of budding yeast genomes and proteomes are currently publicly available. While I by no means suggest that the authors survey the entirety of Saccharomycotina, I think that increasing the depth and sampling of the evolutionary analysis across the subphylum is absolutely necessary for the authors' results and conclusions to be fully supported. Checking gene conservation among a few dozen (30-40) yeast taxa, well-spaced across the budding yeast lineage should suffice to get a more robust picture of which genes truly are young and which are ancient.

We took this point very seriously, and added additional species to our analysis. However, it was not a trivial matter to analyse 30-40 species and to update the analysis to incorporate gene duplication events (as was suggested by reviewer #2). In order to create a systematic scalable method, we set about designing a new bioinformatic strategy to address both the species number and the gene duplication concerns. The new pipeline took both the whole genome duplication and other gene duplications into account, and included many more species in the budding yeast sub-phylum such that for each of our three species examined, we had different sets of three species (each) for genus, Clade, Subphylum and Phylum (the same for all three) (Fig 3, Supp Fig 4, Supp Fig 7). This provided a much more robust view of where in evolutionary terms enrichment occurred. We are grateful that the reviewer pointed us in this direction as our conclusions were strengthened by this analysis.

The approach that is summarised above is described in the results section of the revised manuscript lines 212 – 227 and in the supplementary methods under the heading “Gene Sorting Analysis Pipeline for Figures 2, 3, and Supplementary Figure 7”. The data are shown in Fig 3, Supp Fig 4, Supp Fig 7).

Other than this, I do not have any major or minor issues with the paper, except maybe for the use of the term "unenriched for", where I think "depleted for" would be more appropriate.

We no longer use the term : "unenriched for"

Reviewer #2 (Remarks to the Author):

1 – What are the major claims of the paper? Are they novel and will they be of interest to others in the community and the wider field? If the conclusions are not original, it would be helpful if you could provide relevant references.
I like this paper and the idea of testing stress response in organisms that are relevant and in settings that sort of mimic what the organisms meet in biotechnological and industrial production settings. The main claim of this paper is that the results show that "few significant changes were shared between [yeast] organisms after adaptation to the same stress. … [G]enes that were shared broadly amongst eukaryotes (ancient genes) were less responsive to stress than genus or species-specific (young) genes for each of the yeasts in response to each stress." The results support this analysis and it is consistent with previous results (see the references below for some examples).
A minor (to me) claim is that "This work presents a novel approach to understanding stress gene expression and finds that efforts to engineer stress tolerance could benefit from consideration of the evolutionary age and adaptation rate of target genes."

We think that there is very substantial novelty both in the methodology that we develop for identifying evolutionarily young genes and in the findings themselves. The data presented in Figure 1 in our work suggests recent gene origin and recent gene duplication correlate with differential expression. Thus, we were inspired to rethink gene age by creating a new sorting method that is an updated version of phylostratigraphy, which considers both gene age (like phylostratigraphy) and gene duplication timing (not addressed by phylostratigraphy). This is a novel approach and it enabled us to specifically focus on evolutionary age of the genes as a possible "overarching theme". The subsequent global analyses that were made possible by this method established the importance of gene age as a major predictor of the likelihood of being involved in long-term stress response, thus adaptation.

I am not entirely sure about the novelty of this approach. It is established that stress responsive genes often evolve by gene duplication followed by divergence. Example are analyzed in https://royalsocietypublishing.org/doi/10.1098/rsos.171198 or https://doi.org/10.1371/journal.pgen.1000581. This means that stress- responsive genes tend to be younger genes. This was also found in an analysis of yeast genome, where genes that change their expression during stress response are enriched in unknown function and depleted from genes involved in complexes (https://doi.org/10.1371/journal.pcbi.1000674). Further, comparative analyses of bacterial genomes suggest that evolution of stress response genes tends to be network-modular (to different degrees) and that innovation tends to center around the "sensor" genes rather than around "transmitter" or "actuator" genes (https://www.pnas.org/content/105/21/7500). Thus, in my mind it is already established that "engineer[ing] stress tolerance could benefit from consideration of the evolutionary age and adaptation rate of target genes."

We can understand why the reviewer would question whether it might have been already established that evolutionary age and adaptation would matter. Indeed, our data and those in the studies cited by the reviewer in plants and yeast are consistent with each other. Those studies separately looked at duplicated genes (plants) and short term transcriptional responses (yeast) whereas we focused on (evolutionary) gene age in yeasts spanning hundreds of millions of years of evolution and long-term adaptive responses . Knowing the results of our study, some findings from earlier studies can be interpreted to predict some of our findings, but in fact none of those studies discovered that evolutionary age was the underlying driver and so those conclusions would be formed somewhat *a posteriori* of the knowledge that we generated. For example, Supplementary Figure 1 shows that 20% of the genes we detected by RNA-seq were genes of unknown function in *K. marxianus*, which would be predicted to be enriched for stress responsiveness. Indeed, these genes are two fold enriched versus genes of known function for stress responsiveness (Supplementary Figure 1B). However, our gene age analysis identifies the 4% of the genome that represent the youngest gene group, which show a 3-5 fold enrichment for stress responsiveness (Figure 3B). Thus, we would argue that although these

groups share many of the same genes, gene age is the more robust predictor of stress differential expression.

Although we had not cited the specific studies mentioned, we had not ignored earlier and cited several studies in our manuscript where we can see how our data are compatible with other previous studies (for different parts of the story).:

- Lines 308 – 310 - *Previous works have shown that low expression and non-essentiality correlate with increased adaptation rates*[32,33]*, suggesting that young genes could adapt more rapidly compared to ancient genes.*
- Lines 362 – 370 - *Previous works have linked some young genes to species and genus-specific adaptations, including movement on the surface of fast water in Rhagovelia water striders*[37]*, HIV-1 resistance in owl monkeys*[38,39]*, and the concurrent evolution of antifreeze proteins in several species*[40–42]*. Antifreeze protein genes are well-studied examples of young genes that arose via de novo gene origin events between 13 and 18 million years ago in codfishes and are present at variable copy number in some species*[43]*. Concurrently, the psychrophilic yeast G. antarctica, has evolved to encode nine antifreeze protein genes whose expression levels are induced by exposure to cold*[15,44]*.*

One of the most powerful aspects of our revised manuscript is the number of species that are included – essentially spanning the entire budding yeast subphylum. The findings that we and the reviewers refer to from other previous studies came from different individual species. We have been able to demonstrate consistent findings across hundreds of millions of years of evolution and this provides a strong degree of confidence in the robustness of the findings.

A final important point about novelty is that we present a single set of gene sorting rules that are sufficient to enrich for stress-responsive genes in multiple diverse species exposed to multiple stressors. This consistency suggests that ours is a robust tool for probing broad evolutionary trends and our pipeline will enable enrichment for influencers of niche-specific phenotypes across the subphylum.

2 - Is the work convincing, and if not, what further evidence would be required to strengthen the conclusions?
I have some methodological doubts about the work. While I realize that comparing 1:1:1 orthologs makes the analysis simpler, it also masks the nuances.
In addition, the methods should be more detailed, as in many cases I could not understand the specifics of how the analysis was done.
I remark that I am not doubting the analysis, I just think it should be explained in a way that one could reproduce it without having to guess the intermediate steps.

This explanation is no longer presented in the work. To clarify how the sorting method works we included a flow diagram of the sorting paradigm (Supplemental Figure 4), re-worded the brief version of the description (lines 213-228), and added additional information about sorting to the supplemental methods (**Gene Sorting Analysis Pipeline for Figures 2, 3, and Supplementary Figure 7).**

Also, by analyzing 1:1:1 orthologs you are working against yourselves. If you are correct, then stress response genes should be enriched in genes for which there are no orthologs in the other yeast or for which there has been duplication, and the duplicated genes could evolve and gains new biological function. I would add this analysis to you work.

This comment was fundamental to updating the entire manuscript that is being submitted now. As the updated version of Figure 1C illustrates, both duplicated genes of ancient origin and unshared genes are enriched for stress responsiveness. Thus, the reviewer was correct that our initial analysis, and state of the art approaches like Phylostratigraphy, are weakened by their disregard for duplication and divergence. Both our previous method and Phylostratigraphy lump all members of a gene family into

the most ancient age group with a homolog, which overemphasizes the timing of a *de novo* gene birth and fails to consider duplication and divergence as a mode of functionalization. The comment above spurred us to develop an improved method that is the first of its kind, which predicts gene age by considering both gene duplication and *de novo* gene birth in the same pipeline. Ultimately, both considerations were integrated into the orthology-based approach presented in this manuscript, which vastly improved the results implicating the enrichment of young genes amongst stress responses. To accomplish this, we separated single and multi-copy genes at the beginning of our gene age prediction analysis, which is described in lines 212 – 227 of the revised manuscript.

3 – On a more subjective note, do you feel that the paper will influence thinking in the field? Please feel free to raise any further questions and concerns about the paper.

This is a question the journal should either define better or entirely remove from your reviewer instructions. I have grown old enough to realize that influence in the thinking of a field tend to be more tied to luck, networking and general politics than to a specific paper or set of results.
Hence, the editors, as gatekeepers are much influent in determining/predicting/setting this up than the reviewers.

No comment from authors

4 – Specific comments and suggestions:
Page 3: "Other yeast species adaptations that make them well-suited for production of specific biomolecules, but have been difficult to engineer until the recent emergence of CRISPR/Cas9"
Unclear. Please rephrase and clarify what you mean.

Lines 65 – 67. This is rephrased for clarity.

Page 3: "Analysis of 2,903 orthologous protein coding genes found that few significant changes were shared between these organisms after adaptation to the same stress."
At this stage the reader questions why only analyzing 2 903 orthologous genes and not the entire genomes. Only latter the reason is given. I would rephrase this in order not to put the reader in a mindset "Why not look at them all?" from the beginning.

This is no longer an issue in the revised approach and that section is not present.

Page 4: "To assess the fidelity of ortholog predictions, protein complexes and enzymatic processes that are expected to be conserved amongst budding yeasts were searched for amongst the core proteins."
Please describe how this comparison was done in detail in methods.

This section refers to a comparison of our results with a previous publication that characterized single copy genes that are conserved amongst yeasts and emerged prior to the divergence of modern yeasts and mammals. We have updated lines 159-161 to clarify this statement.

Page 5: "Core Genes and Proteins are Unenriched for Stress-Responsive Expression"
This should be "Core Genes and Proteins are depleted for Stress-Responsive Expression"

We do not use this phrase any longer

Page 5 "A separate gene grouping analysis was carried out for each yeast to include relatives that had similar estimated times of common ancestor divergence. Orthologous gene predictions were used to sort each protein, and its coding gene into a single bin based on the most distant ancestor with a predicted orthologous protein (Supplemental Figure 3a and Supplemental Figure 3b)."
Unclear what exactly was done here. Please expand and clearly explain the analysis, controls and

comparisons.

This explanation is no longer presented in the work. To clarify how the sorting method works we included a flow diagram of the sorting paradigm (Supplemental Figure 4), re-worded the brief version of the description (lines 213-228), and added additional information about sorting to the supplemental methods (**Gene Sorting Analysis Pipeline for Figures 2, 3, and Supplementary Figure 7).**

Page 6: “Together, these findings show that the young genes were enriched for stress-responsiveness compared ancient genes for each yeast species in this study after adaptation to each stress condition.” Please correct sentence

Sentence is no longer present.

Page 6: “As the species chosen for this analysis span much of the diversity of the budding yeast subphylum, these results may be indicative of a conserved stress adaptation mechanism amongst budding yeasts.”

Sure, and the mechanism is likely to be gene/module duplication followed by divergent evolution, which is common to other organisms (see references above).

As discussed above, the analysis and conclusions have been greatly strengthened in the revised manuscript. Gene duplication is part of the story, but there are many other non-duplicated evolutionarily young genes also now implicated in long-term stress adaptation and thus niche specialisation in this study. This expands the concept significantly beyond what was previously known or postulated

Page 6: “Stress shock and stress adaptation exhibit different gene expression changes (Supplemental Figure 1b and 1c), which may be indicative of distinct response mechanisms.”
Supp. Figs 1b (especially) and 1c do not really allow me to see this. There is a large commonality between the two sets. If I am right, a plot taking all genes from the two sample being compared and plotting change-fold or (quantile of change-fold) should have a strong correlation/Adjusted R squared.

This comparison is not made in the revised manuscript

Page 6: “experiments from Hughes Hallett 2014 showed”

Line 381. The sentence referring to that paper (reference 17) is changed

Please fix.

Page 6-7: “In contrast, the stress adaptation experiments from this report showed young genes were enriched amongst both upregulated and downregulated genes in response to osmotic and temperature stress (Supplemental 7c and 7f). These results suggest that stress shock and stress adaptation gene expression differ in relation to gene age, especially amongst downregulated genes.”
I think the authors could propose possible interpretations for this observation. Results from https://doi.org/10.1371/journal.pcbi.1000674 and the reference you give show that early stress response is enriched in “young genes”. If there is a second set/module of stress responsive genes responsible for long term adaptation, then it would be plausible that the early response module is down regulated (hence enrichment in younger genes in the set of down-regulated genes during long term response) and the later adaptation module is upregulated (hence enrichment in younger genes in the set of down-regulated genes during long term response). If this interpretation is correct, the set of downregulated young genes in long term response should have a large overlap with the set of young genes upregulated in the early response. This can be checked in your datasets.

Analysis of this and other points led us to focus on comparing stress shock and long term stress tolerance in a separate manuscript. Ultimately, this is a rich topic that did not seem possible to properly analyse in a supplemental figure as was attempted in the previous version.

Page 7: “Young Genes are Less Expressed, Shorter, Less Likely to be Essential, and Accumulate More Sequences Changes than Ancient Genes.”
I don’t think results in this section are entirely new. See the references above.

This point is covered in the discussion above

Page 10: Please correct typos
“media for 24hours in”; “listed in Supplemental 1A.”; “Cultivations for were performed”

Corrected (line 404 and 411)

Page 10: “Steady state growth was defined as less than 5% deviation in biomass dry weight.” Please describe how this was assessed.

The basic method was that developed by the Delft group for studying yeast physiology (e.g. van Hoek et al., 1998,. AEM 64: 4226–4233). The dry weight was measured every 24h after at least 5 volume changes of the medium in the vessel after the switch from batch to continuous culture. 10ml volumes were sampled and dried on pre-weighed filters. Samples were tested in duplicate. If there was not change (within the permissible deviation) for three days in a row, it was determined that the chemostat was in steady state.

Page 11: “MS data are available online on public databases via the PRIDE39 repository with the dataset identifier PXD011426 and PROTICdb37,40 or via the following URLs:
http://moulon.inra.fr/protic/chassy_kluyveromyces
http://moulon.inra.fr/protic/chassy_yarrowia
http://moulon.inra.fr/protic/chassy_saccharomyces”
Please make sure these datasets are public. I tried to access them to make sure they were accessible and they were behind a password.

All the data (proteomics and transcriptomics) are now available without passwords (Lines 456 – 460) using the database links there and in the methods section.

Reviewer #1 (Remarks to the Author):

The authors have addressed my concerns completely with this revised focus/analysis.

Reviewer #3 (Remarks to the Author):

I am pleased to see the revised manuscript and the expanded methodology and broader focus, it certainly looks better than the original one.

I believe that the revised manuscript addressed most, if not all of the specific comments made by Reviewer #2 (or they became irrelevant in the new version). The new analysis that the authors included, which stemmed from a comment by Reviewer #2 does cover more ground and looked at stress-responsive gene evolution in a more robust and precise manner. As for the question of novelty of the submitted work, the authors do acknowledge earlier papers and I think they present a potent novel methodology of high evolutionary breadth and significance.

Reviewer #4 (Remarks to the Author):

This study analyzes gene expression changes upon stress in different yeast species and concludes that "Stress-Induced Expression is Enriched for Evolutionarily Young Genes in
Diverse Budding Yeasts". The concept itself had been previously proposed but this study presents a nice example including in species where the relationship between stress and young genes had not been investigated before and where it is particularly important given their biotechnological relevance.

I was asked specifically to inspect the methods introduced by the authors to classify genes in different evolutionary age categories, so this is the only focus of my review. The method employed by the authors is sound in my opinion. It is very coarse grained but this is not an issue for the analyses presented here. There is one issue though, not really with the method itself but with the interpretation of the results. When the authors assign genes to their age groups, it is based on the fact that they could not detect homologues outside of the corresponding branches. The use of orthofinder, which is blast based and relies on annotations, as well as the fact that the phylogenetic tree was sparsely sampled, necessarily mean that some homologues will be missed. This could be somehow remedied by adding a blast against nr to find more distant homologues. Yet, the intrinsic limitations of blast based methods and reliance on annotation cannot be fully remedied. Hence, the analyses presented here do not allow to conclusively determine that the young genes have emerged in a given branch (whether de novo or by duplication). My suggestion to the authors would be to slightly rephrase their conclusions and indicate that the genes they call "young" have branch specific sequences, which may or may not correspond to branch specific evolutionary emergence. In my view, clarifying this would go a long way increasing the rigor of the study, without affecting the conclusions and possible applications for biotechnology at all.

I would also request that the authors provide more details in their methods section including the version numbers for reference annotation, parameters etc.

REVIEWERS' COMMENTS:

Reviewer #1 (Remarks to the Author): - no author reply necessary

The authors have addressed my concerns completely with this revised focus/analysis.

Reviewer #3 (Remarks to the Author): - no author reply necessary

I am pleased to see the revised manuscript and the expanded methodology and broader focus, it certainly looks better than the original one.

I believe that the revised manuscript addressed most, if not all of the specific comments made by Reviewer #2 (or they became irrelevant in the new version). The new analysis that the authors included, which stemmed from a comment by Reviewer #2 does cover more ground and looked at stress-responsive gene evolution in a more robust and precise manner. As for the question of novelty of the submitted work, the authors do acknowledge earlier papers and I think they present a potent novel methodology of high evolutionary breadth and significance.

Reviewer #4 (Remarks to the Author):

This study analyzes gene expression changes upon stress in different yeast species and concludes that “Stress-Induced Expression is Enriched for Evolutionarily Young Genes in Diverse Budding Yeasts”. The concept itself had been previously proposed but this study presents a nice example including in species where the relationship between stress and young genes had not been investigated before and where it is particularly important given their biotechnological relevance.

I was asked specifically to inspect the methods introduced by the authors to classify genes in different evolutionary age categories, so this is the only focus of my review. The method employed by the authors is sound in my opinion. It is very coarse grained but this is not an issue for the analyses presented here. There is one issue though, not really with the method itself but with the interpretation of the results. When the authors assign genes to their age groups, it is based on the fact that they could not detect homologues outside of the corresponding branches. The use of orthofinder, which is blast based and relies on annotations, as well as the fact that the phylogenetic tree was sparsely sampled, necessarily mean that some homologues will be missed. This could be somehow remedied by adding a blast against nr to find more distant homologues. Yet, the intrinsic limitations of blast based methods and reliance on annotation cannot be fully remedied. Hence, the analyses presented here do not allow to conclusively determine that the young genes have emerged in a given branch (whether de novo or by duplication). My suggestion to the authors would be to slightly rephrase their conclusions and indicate that the genes they call “young” have branch specific sequences, which may or may not correspond to branch specific evolutionary emergence. In my view, clarifying this would go a long way increasing the rigor of the study, without affecting the conclusions and possible applications for biotechnology at all.

We accept the reviewer’s point, which in a sense is that limitations in current large-scale genomic datasets, annotation and analysis mean that there must always be a degree of uncertainty – for example, until we have every gene annotated in every yeast species, one cannot be absolutely sure with 100% confidence that a gene is unique to a branch. It is appropriate to recognise that such a degree of uncertainty is intrinsic to any large-scale genomics study. We have taken the reviewer’s advice and added text (lines 271 – 276) to acknowledge this reality.

I would also request that the authors provide more details in their methods section including the version numbers for reference annotation, parameters etc.

Version numbers have been added as requested
